# Dynamic Signatures of the Epigenome: Friend or Foe?

**DOI:** 10.3390/cells9030653

**Published:** 2020-03-07

**Authors:** Marta Machnik, Urszula Oleksiewicz

**Affiliations:** 1 Department of Cancer Immunology, Poznan University of Medical Sciences, 60-806 Poznan, Poland; marta1machnik@gmail.com; 2 Department of Cancer Diagnostics and Immunology, Greater Poland Cancer Centre, 61-866 Poznan, Poland

**Keywords:** epigenetics, evolution, KRAB–ZNF, transposable elements, cancer

## Abstract

Highly dynamic epigenetic signaling is influenced mainly by (micro)environmental stimuli and genetic factors. The exact mechanisms affecting particular epigenomic patterns differ dependently on the context. In the current review, we focus on the causes and effects of the dynamic signatures of the human epigenome as evaluated with the high-throughput profiling data and single-gene approaches. We will discuss three different aspects of phenotypic outcomes occurring as a consequence of epigenetics interplaying with genotype and environment. The first issue is related to the cases of environmental impacts on epigenetic profile, and its adverse and advantageous effects related to human health and evolutionary adaptation. The next topic will present a model of the interwoven co-evolution of genetic and epigenetic patterns exemplified with transposable elements (TEs) and their epigenetic repressors Krüppel-associated box zinc finger proteins (KRAB–ZNFs). The third aspect concentrates on the mitosis-based microevolution that takes place during carcinogenesis, leading to clonal diversity and expansion of tumor cells. The whole picture of epigenome plasticity and its role in distinct biological processes is still incomplete. However, accumulating data define epigenomic dynamics as an essential co-factor driving adaptation at the cellular and inter-species levels with a benefit or disadvantage to the host.

## 1. Introduction

Genetic mutation and epigenetic alterations determine species diversity and adaptability to changing environmental conditions. They may also have a negative effect, if the mutation or epigenetic modification hampers the expression or functioning of an essential gene, thus leading to various diseases. While genetic changes remain throughout the lifetime of an organism, epigenetic events are reversible and occur at a much faster rate than genetic changes, thus providing more flexible mechanisms of adaptation [1]. Genetic mutations are limited to the DNA sequence. In contrast, the epigenomic status of a single cell depends on a wide array of multilayered, interconnected events that include chemical modifications of DNA and histones, 3D chromatin structure, as well as the expression profile of chromatin modifiers, non-coding RNAs and prions [2]. In general, epigenomic states define the packaging of chromatin in the nucleus and its accessibility to other factors involved in various DNA activities, including transcription [3], damage response and repair, recombination, and replication [4]. 

Epigenomic alterations were initially linked to the developmental processes [5]. Indeed, the most dramatic epigenomic shifts occur during embryogenesis. Upon fertilization, the gametes’ genomes become epigenetically reprogrammed to enable zygotic genome activation and totipotency. With subsequent cell divisions and the onset of differentiation, the cells progressively acquire new epigenomic profiles characteristic for a given cell lineage with specialized function and limited potency. These developmental epigenomic modifications appear in an ordered, programmed fashion [6]. Somatic cell reprogramming to induced pluripotent stem cells is also dependent on massive alterations within the epigenomic profile of the cells [7,8]. Successful reprogramming reversely mirrors early developmental processes and requires the elimination of epigenetic barriers [9,10] to acquire open chromatin state that is characteristic to stem cells [8,11]. The maintenance of the epigenome becomes more error-prone with age, thus leading to so-called “epigenetic drift”—accumulation of epigenomic aberrations. For instance, during aging, DNA becomes globally hypomethylated with some local hypermethylation, mainly within gene promoter regions. Additional alterations in a histone code and chromatin structure lead to increasing epigenome instability. Many studies show that these accumulating epigenomic alterations could be correlated with age-related diseases, including neurodegenerative disorders, atherosclerosis, osteoarthritis, and even cancer [12]. The majority of cells in the organism contain the same genetic code, but their phenotypes differ substantially. The acquisition and maintenance of a particular cell phenotype depend mainly on the stable expression of tissue-specific genes. Importantly, the gene expression profile is orchestrated through various epigenetic mechanisms affecting chromatin accessibility for transcription factors and the regulatory function of non-coding RNAs (ncRNAs) [3]. Dynamic distribution of epigenetic marks at specific time points defines cellular phenotype (functionality, differentiation state, and age) [13]. Certain epigenomic signatures might be reversible and very short-lived (e.g., chromatin changes occurring in response to DNA damage) [4]. Other modifications might be inherited through mitosis or meiosis. A spontaneously occurring epigenetic mark passed over to the progeny upon cell division is known as epimutation. In specific cases, the inherited epimutation might last in only one generation (i.e., intergenerational inheritance). In other, it may become a persistent change that is passed throughout the lifespan of a particular cell lineage or may be inherited transgenerationally as a driver of evolution [14,15]. 

Here, we present the current models describing the mechanisms, as well as genetic and environmental factors that shape the epigenomic profile, particularly in human cells. We concentrate on the positive and negative consequences of epigenetic plasticity that are associated with improved adaptation to changing conditions and/or may evoke pathological processes as observed, e.g., in various metabolic syndromes and carcinogenesis. Our primary focus of attention is DNA methylation and, to a lesser extent, histone post-translational modifications, which reflect the fact that from the plethora of epigenetic alterations, these two are studied most often. Firstly, we demonstrate the causes and effects of environmental stimuli on inter- and trans-generational epi-mark inheritance. Secondly, we portray the co-evolution between genetic and epigenetic signatures driven by transposable elements (TEs) and Krüppel-associated box zinc finger genes (KRAB–ZNFs). The view on paired TE and KRAB–ZNF co-evolution emphasizes the “arms-race” and domestication mechanisms contributing to higher sophistication of transcriptional control. Thirdly, we report the concepts on the genetic and epigenetic co-evolution processes associated with clonal heterogeneity and expansion occurring during cancer development. These three examples represent different but partially overlapping, context-dependent mechanisms responsible for the evolution of epigenomic signatures.

## 2. Molecular Mechanisms behind the Inheritance of DNA Methylation and Posttranslational Histone Modifications

DNA methylation is the best characterized epigenetic modification to date. In animals, the addition of a methyl group (-CH3) occurs on cytosines residing mainly within a cytosine–guanine (CpG) context symmetrically on both strands. The CpG-dense regions, known as CpG islands (frequently occurring within gene promoters), are usually hypomethylated, whereas single CpGs interspersed within the genome are methylated [16]. During replication, the newly synthesized DNA strand lacks methylation. Hemimethylated DNA recruits DNA methyltransferase 1 (DNMT1) that catalyzes the transfer of the methyl group onto cytosine. Such template-based methylation ensures faithful inheritance of DNA methylation profile upon cell division [17].

Post-translational modifications of histones are much more complex than DNA modifications. The combination involves three factors: histone (H1, H2A, H2B, H3, and H4) or its variant (e.g., H3.3, H2A.Z), residue and its position (e.g., lysine (4, 9, 27), serine, threonine, arginine, proline), and molecular modifications (e.g., methylation, acetylation, phosphorylation, ubiquitination, citrullination, ADP-ribosylation). Histone modifications influence 3D histone conformation or its electric charge, thus affecting the histon:histon or histon:DNA interactions. Moreover, they might create a docking or repelling site for different factors involved in transcription or chromatin structure regulation. Post-translational modifications on histones and other epigenomic signatures create a communication platform to mark various genomic regions (e.g., active promoters or enhancers) and mediate specific DNA activity [18]. There are still some open questions about the molecular mechanisms responsible for the precise inheritance of histone modifications. The data indicate that the precision may depend on the enzyme and substrate (e.g., folate, phosphate) availability [19,20,21,22], as well as ncRNA and DNA sequence that guide or recruit epigenetic modifiers to specific location [23,24,25]. The exact molecular mechanisms driving the heritable transmission of histone modifications are still incompletely understood. The current model indicates that during replication, the parental, modified histones remain in the nucleus. Upon replication, a nucleosome is split into two H2A–H2B dimers, and an H3–H4 tetramer (or two H3–H4 dimers depending on the histone variant composition) [26,27]. Old H3–H4 interacting units are randomly incorporated into both daughter strands, while the empty spaces are filled with newly synthesized histones (unmodified or transiently acetylated). The deposition of H3–H4 tetramer is followed by the assembly of proximal and distal H2A–H2B dimers. Thus, it is expected that the H3–H4 complex plays a major role in the epigenetic inheritance of histone post-translational modifications. The post-translational modifications on the parental histone constitute so-called “epigenetic memory”—they serve as a template that enables the introduction of similar alteration onto newly deposited histone [28,29,30]. The parental modification recruits a protein complex with subunits that recognize, maintain, and introduce the same mark on the nearby nucleosomes. Thus, the presence and local concentration of a given epigenetic modification within a specific genomic region are crucial for the robust inheritance of this mark during cell division [28,29,30,31,32,33]. Of note, the parental histones with repressive modifications are re-deposited locally to the same DNA domains, while the histones with active marks lose their local distribution [31]. These observations underpin the complexity of the mechanisms implicated in the epigenetic inheritance of histone modifications.

## 3. Germline Inheritance of Epimutation

Some molecular mechanisms behind the inheritance of epigenetic marks are relatively well-characterized, but others, as described above, are still poorly understood. It is well established that epigenomic states are transmitted through mitosis and meiosis. Therefore, there is some possibility that epimutation, whether spontaneous or induced by genetic or environmental events, may be transferred to progeny, and as such, may affect evolutionary processes. Nevertheless, the influence of germline epimutation on evolution remains debatable. Epigenetic changes are frequently coupled with genetic variability [34,35,36,37], which poses questions about the driver function of epimutation. It is challenging to develop an appropriate research model for studying epimutation-driven transgenerational evolution. Several barriers should be overcome for the epimutation to be maintained in the population. The epimutation must persist genome-wide epigenomic changes occurring during developmental processes: in germlines (e.g., histone exchange for protamines in sperm), after fertilization, and during lineage commitment. Notably, the epigenome undergoes two waves of DNA demethylation: the first at the pre-implantation stage, and the second during primordial germ cells (PGCs) development. Moreover, the epimutation should serve as a permanent change in the gene expression profile that reinforces potentially more advantageous phenotype [14]. 

Despite massive chromatin reorganization, the epigenetic mechanisms required for the establishment of an appropriate developmental transcriptome creates a window for the inheritance of both programmed and spontaneous epigenetic change. Oocyte retains histones together with their epigenetic traits [38]. In sperm, individual histones, their variants, and post-translational modifications, as well as 3D chromatin structure, are resilient to the replacement with protamines [22,39,40]. Moreover, an increasing number of studies provide evidence that ncRNAs are also subject to germline inheritance, whereas the alteration in their expression profile induced by environmental cues may affect the phenotype in progeny [14,41,42]. Published data also indicate that some CpG sites may remain methylated despite both developmental demethylation waves [43,44,45,46]. Genomic imprinting is the best-known example of epigenetic inheritance that endures global demethylation in an embryo. The parental imprints are erased during PGCs formation, and the new pattern that reflects the sex of an embryo is established [44]. A growing number of data indicate that the imprints are not the only epi-marks that can be inherited. For instance, Hackett and colleagues demonstrated that rare CpG sites might be resilient to global methylation erasure occurring during PGCs formation [43]. More examples of the novel epigenetic features retained in progeny are provided in Section 4 and Section 5 below. 

As described above, molecular inheritance mechanisms may allow for the transmission of a newly acquired epigenetic trait to the progeny regardless of its origin. However, the stable inheritance of epimutation during evolution is regarded as a relatively rare event. Interestingly, the in-depth analysis of Arabidopsis methylome demonstrated that genetic mutations occur less frequently and are much more stable than epimutations. Backward mutations were estimated to occur at the frequency of 16-times lower than forward mutations, while backward epimutations occurred three times faster than forward epimutations [47]. Such a phenomenon might be explained by the fact that a single epigenetic modification does not play solo, but rather it acts as a player in an orchestra of a variety of epigenomic changes that jointly affect chromatin status. Thus, newly occurring epimutation may be erased during the next cell division because the regional chromatin state may serve as a template for the re-establishment of a given epigenetic mark. The synchronized occurrence of cooperating epimutations may, therefore, enhance the stability of a new phenotype. Moreover, genetic changes that affect epigenetic alterations may also stabilize novel epigenetic features.

## 4. Environmental Influence on Epigenetics

**Figure 1:**
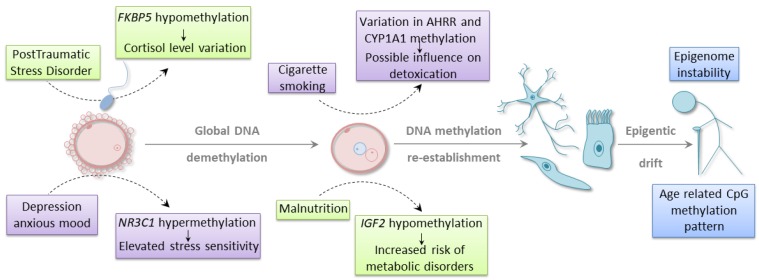
External factors have an impact on the health outcome of an individual. Stressors acting on parents-to-be can influence the well-being of an offspring: stress response can be altered in children of fathers that experience post-traumatic stress disorder or of mothers with a tendency to depression/anxious moods. In utero exposition to harmful factors can also change the health of an offspring: malnutrition can be a cause of *IGF2* hypomethylation, thus an increased risk of metabolic disorder, and cigarette smoking during pregnancy may alter the detoxication processes in a child.

It is well established that external stimuli, such as environmental conditions, diet, or lifestyle choices, may affect developmental plasticity and susceptibility to different diseases (Figure 1) [48,49]. In utero exposure to environmental challenges may augment the risk of disease development, also in the next generations through epigenetic mechanisms. However, it is still unclear whether epimutation may, at least in some instances, act as a sole driver of evolutionary adaptation or a parallel genetic alteration is also required for mediating an epigenetic effect. Many of the published data that demonstrate the correlation between environmental factors and epigenetic changes do not take into account potential confounding factors, such as genetic mutations and polymorphisms. Another limitation of many association studies is the lack of functional evidence that would link epigenetic changes to gene expression profile and phenotypic alterations. In order to resolve the controversies around the relationship between environment, epigenetics, and evolution, it is crucial to employ a more comprehensive methodology. Such an approach should consider the complexity of environmental factors, the possibility of occurrence of genetic and epigenetic changes, and the manifestation of associated phenotype. It remains to be established how the genetic and epigenetic alterations are interconnected in terms of cause and effect paradigm. Nevertheless, environmental stimuli may shape the epigenomic landscape regardless of whether this effect is direct or indirect through genetic events. The current section will focus on the studies that demonstrate such a relationship, suggesting that epigenetic alteration may participate in a dynamic adaption in response to environmental challenges.

One of the frequent analytical models is the evaluation of the epigenetic profiles in the progeny of the parents exposed to a particular lifestyle or environmental conditions. For example, Joubert and colleagues performed genome-wide methylation analysis in cord blood of the children whose mothers smoked during pregnancy [50]. They identified 26 differentially methylated regions (DMRs) associated with maternal smoking. DMRs were located in 10 genes, and two of them, *CYP1A1* and *AHRR*, are known to participate in the signaling pathways involved in the elimination of toxic compounds from tobacco smoke. Four of the identified CpGs were located in a region upstream of *CYP1A1*, and their methylation level positively correlated with the level of nicotine metabolite measured in the mother’s blood. Another four CpGs resided within the coding region of *AHRR*. The methylation level of one of the CpGs inversely correlated with the nicotine metabolite level [50]. Interestingly, a similar pattern of DNA methylation for *AHRR* was observed in adult smokers, where increasing methylation of the same CpG was associated with decreasing *AHRR* gene expression [51].

A mother’s mental state may also significantly affect the health of the future child. Certain studies showed high methylation level of glucocorticoid receptor gene, *NR3C1,* in cord blood from the newborns whose mothers experienced depression or anxious mood during pregnancy [52,53]. *NR3C1* is a crucial regulator of the HPA (hypothalamic–pituitary–adrenal) axis. The analysis demonstrated that hypermethylation within the CpG-rich region of *NR3C1* was associated with an increased level of cortisol, which was indicative of altered stress sensitivity during infancy [53]. Even preconception parental trauma was shown to influence the epigenetic outcomes in children. The cytosine methylation level of another gene involved in glucocorticoid receptor regulation (*FKBP5*) was measured in Holocaust survivors and their adult offspring [54]. Altered methylation was observed in both groups in comparison to age-matched, unexposed controls. The same site was hypermethylated in a parent cohort and hypomethylated in their children. The observed hypomethylation was negatively correlated with the wake-up cortisol level in offspring. The hypomethylation was not associated with other examined confounding factors, which underlines the influence of parental stress exposure on the emergence of the epigenetic trait [54].

The growing number of evidence indicates that dietary conditions of a parent-to-be may affect the epigenomic landscape leading to a phenotypic shift in children’s or even grandchildren’s health. Indirect evidence of such phenomenon comes from historical periods of human malnutrition, during which large populations were exposed to famine. The descendants of parents who experienced the Dutch Hunger Winter accumulated negative traits through changes in the DNA methylation levels within different genomic regions [55,56,57]. Tobi and colleagues conducted a reduced representation bisulfite sequencing (RRBS) profiling of 24 individuals exposed in utero to severe calorie restriction and their unexposed siblings. They found 181 genomic regions whose differential methylation was associated with prenatal malnutrition. The majority of these regions were hypermethylated. Several identified DMRs mapped to the genomic areas involved in developmental and metabolic processes [56]. Previous observations by the same research group demonstrated as well that one of the consequences of prenatal exposure to famine is hypomethylation of *IGF2* locus. Lowered methylation of *IGF2* was related to an increased chance of metabolic diseases in infants [55]. The parent malnutrition also led to higher BMI, elevated levels of cholesterol and LDL, and a higher risk of neurological disorders in the adult life of the progeny [58]. Additionally, small but significant differences in DNA methylation of *IGF2* [46], as well as other imprinting control regions [45], were also found associated with parental obesity. As no functional characterization of the infants was performed, it is unclear whether the observed small changes in DNA methylation level may have any effect on the expression of tested genes or the phenotype of newborn babies [45,46]. One of the nutrients essential for the appropriate execution of the developmental program is folate, as it contributes to the metabolic pathways involved in DNA synthesis, as well as cytosine and histone methylation. Thus, folate insufficiency has a detrimental impact on genomic and epigenomic stability. Indeed, several reports indicate that the epigenetic instability occurring during gamete formation due to inadequate folate availability [19] or metabolism [20] is transferred to progeny. These epigenetically inherited modifications were shown to contribute to developmental defects [19,20], even over several generations [20]. It is worth mentioning that the population studies presented above are considered quasi-experimental. It is difficult to rule out other effects that may influence tested cohorts. Nevertheless, all of the above findings indicate that epigenetic changes may serve as a dynamic adaption in response to changing conditions. Moreover, several animal studies provide empirical support for these observations. The consequences of improper parents’ diet, as well as stress-mediated alterations that can affect the offspring in an epigenetic-mediated fashion, are well documented [59,60,61,62]. 

The observations mentioned above indicate that many environmentally induced epigenetic traits may have an adverse effect on human health. In certain cases (e.g., malnutrition), the negative impact on progeny well-being may result from the disappearance of severe external conditions. In such a scenario, the epigenetic change is no longer required and might be erased in the next generation. On the other hand, environmental pressure can also contribute to the development of new epigenetic features that will allow for better adaptation, specifically, to endure the continuous challenge. For example, changes in DNA methylation status was shown to be involved in persisting lactose tolerance across different populations worldwide. The majority of mammals lose the ability to digest lactose after weaning, which suggests that epigenetic changes contribute to the shift in phenotype. In some human populations, lactase—the enzyme responsible for cleaving lactose into monosaccharides—is highly active throughout the whole life due to the polymorphisms within the lactase gene (*LCT*) enhancer [36]. High-throughput methylome profiling identified DMRs in the *LCT* promoter and enhancer, whose hypermethylation correlated with reduced lactase expression and activity, and consequently, with lactase non-persistence [36,63]. Of note, DMRs were also highly associated with known *LCT* polymorphism, suggesting the genetic influence on the epigenetic trait. However, the methylation level within the promoter and enhancer outperformed the genotype in the phenotype prediction test, which was particularly evident for SNP heterozygotes [36]. Another epigenetic trait associated with human adaptation is the evolution of molecular defense mechanisms. Interesting epigenetic alterations were observed in the nomadic population of Oromo people who migrated to the Ethiopian highlands. Methylation profiling with 27 K microarrays identified a few DMRs distinguishing high- and low-landers. The alterations in DNA methylation were associated with the genes involved in hypoxia response and HIV infection (i.e., *APOBEC3G*, *MT1G*) [64], suggesting that epigenetic mechanisms may contribute to evolutionary adaptation towards external stimuli.

## 5. Epigenetic Signature in Species Evolution

A common approach to assess the impact of epigenomics on human evolution is the comparison between human methylome and the methylomes of our closest relatives. Comparative analysis between humans and chimpanzees demonstrated that DNA methylation profiles are highly similar [65,66]. Pai and colleagues interrogated the methylomes of several human and chimpanzees samples that represented the heart, liver, and kidney tissues [66]. Utilizing human methylation array, they found that the DNA methylation profile was highly conserved between analyzed species. Interestingly, observed variation was higher between various tissue types than between species. Gene Ontology analysis revealed that species-specific and tissue-conserved DMRs were enriched in terms related to developmental processes and tissue-specific biological functions. The data demonstrated as well that the inter-species divergence in the methylation within promoter regions partially accounts for the differences in gene expression profiles observed between humans and chimpanzees [66]. Further NGS-based DNA methylation profiling of the prefrontal cortex showed that human promoters and genes bodies have an overall lower methylation level than corresponding chimpanzee regions [67]. Promoter hypomethylation correlated with higher expression of the majority of analyzed genes in the human cortex. Interestingly, DMRs frequently resided within the promoters of the genes implicated in disease development. Specifically, hypomethylated human promoters were associated with cancer, as well as neurological and psychological disorders [67]. In another study, Hernando-Herraez and colleagues analyzed CpG methylation patterns from the blood samples obtained from humans and other primates (including chimpanzee, bonobo, gorilla, and orangutan) [68]. Comparative analysis identified human-specific methylation patterns in nearly 200 genes, including the genes associated with tissue-specific functions, as well as developmental and neurological processes [68]. High epigenome conservation and differential methylation patterns specific to certain regions between human and great apes were also confirmed by whole-genome bisulfite sequencing of blood samples [34]. This study demonstrated that the majority of human-specific DMRs overlap with repressed promoters, whereas hypomethylated DMRs additionally occur within active promoters and bivalent domains, as well as enhancers distal from the promoter regions. The appearance of human DMRs correlated with the higher frequency of human-specific mutations within predicted transcription factor binding sites. Furthermore, hypomethylated regions were associated with endogenous retroviral sequences. These observations pinpoint the relationship between genetic alterations and epigenomic signature that may co-occur during species evolution. However, the causal connection between genetic and epigenetic events were not established in this study [34]. 

Another approach for the evaluation of the importance of epigenomic profile in species evolution is a comparative analysis between *homo sapiens* and more ancient humans, as well as the analysis between populations of different ethnic origins. A recent study utilized a novel method based on the natural processes of progressive cytosine deamination [69], which allowed to reconstruct DNA methylation maps of the ancient humans (the Neandertal and the Denisovan) in comparison to modern human methylome [70]. While the majority of methylation pattern was highly conserved between analyzed individuals (~99%), around 1100 DMRs were specific to archaic humans. Interestingly, three of the identified DMRs were mapped to *HOXD* locus, a master regulator of limb development [70]. Ancient humans *HOXD9* promoter and *HOXD10* gene body were found hypermethylated when compared to modern humans. Based on these findings, it is tempting to speculate that epigenetic changes played a role in the evolution of human limbs. Other DMRs were mapped to the genes implicated in developmental processes, as well as neurological and psychiatric diseases [70].

Interesting outcomes come from the comparative analysis of various modern populations with different ethnic backgrounds. For example, Heyn and colleagues assessed the global DNA methylation profile in Caucasian-, African- and Han Chinese-American populations [71]. A specific pattern of DNA methylation was able to distinguish individuals based on their ethnicity. Ontology assessment showed that population-specific DMRs were related to the genes associated with distinct biological processes that determine natural human variation, i.e., xenobiotic metabolism and transport, environmental information processing and adaptation, immune response factors, keratinocyte-related genes, and disease susceptibility. Enrichment analysis of DNA motifs indicated that many of the DMRs were related to transcription factors, including hematopoietic factors IRF1 and SPIB. Although the majority of DMRs correlated with genetic variants, around 32% of DMRs (associated mainly with immune response) did not show any connection to genetic background. This suggests that epigenomic modifications may, to a certain extent, impact evolutionary processes independently of genomic variability [71]. Many of these observations stay in agreement with another study conducted on five different human populations [72]. Carja and colleagues were also able to separate distinct populations based on their DNA methylation patterns. Moreover, they found that DNA methylation marks that are specific to a particular ethnic background strongly reflect the genetic variation. Similarly, as in [71], the majority of population-specific DMRs were found outside of CpG islands, mostly in CpG shores, shelves, and "open seas" [72]. Altogether, the findings regarding differences in DNA methylation signatures between various human populations, as well as modern humans and their ancestors, suggest that epigenetic variation may contribute to evolutionary adaptation. The above studies gave evidence that epigenetic modifications may underlie species evolution by regulating the developmental capabilities, psychological functions, and disease susceptibility. Thus, to obtain a more comprehensive portrayal of the phenotypic variation between human populations, epigenomic studies may prove complementary to the currently undergoing intensive genome-wide association studies.

## 6. The Co-Evolution between Transposable Elements and their Epigenetic Repressors

**Figure 2:**
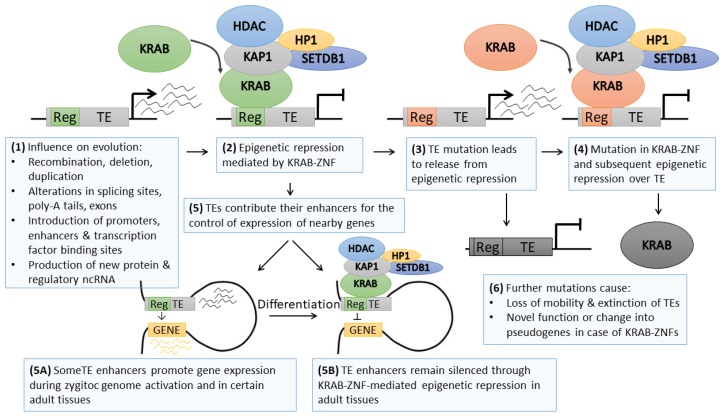
The co-evolution between TEs and KRAB–ZNFs. (**1**) Active TEs may be expressed and retrotransposed to another location in the genome, thus providing novel, functional sites within integration locus. (**2**) A KRAB–ZNF binds to a specific TE and promotes its epigenetic repression. (**3**) Mutation within the TE sequence results in the escape from KRAB–ZNF-mediated epigenetic repression. (**4**) Upon genetic drift, the novel KRAB–ZNF factor recognizes the mutated TE sequence and facilitates its epigenetic inactivation. (**5**) KRAB–ZNFs participate in the domestication of regulatory sequences residing within TE. Upon zygotic genome activation and in certain tissues (**5A**), TEs are de-repressed, and their enhancers are utilized for gene expression regulation. Upon differentiation (**5B**), repression of TEs is maintained by their specific KRAB–ZNFs. (**6**) Further genetic drift in TEs results in their permanent immobilization and extinction. Mutations in KRAB–ZNFs may provide new functions or lead to their degradation to pseudogenes. Reg, regulatory sequence.

One of the interesting examples of cooperation between genetics and epigenetics as common drivers of evolution is the phenomenon of transposable element (TE) silencing by KRAB–ZNF genes (Figure 2). TEs are ancient repeat elements interspersed within the genome that emerged most likely as structural genes that acquired mobility [73] or due to viral infections in the germline followed by consecutive multiplication in the host genome [74]. TEs constitute approximately half of the human genome [75]; however, it is expected that due to the genetic drift, repetitive sequences may be much more frequent (around 2/3 of the human genome) [76]. Their high abundance is related to their mobility—TEs may be cut (transposons) or copied (retrotransposons) from one locus and pasted to another. They are categorized into several classes depending on their structure: autonomous endogenous retroviruses (ERVs) with long terminal repeats (LTRs) and non-LTR long interspersed nuclear elements (LINEs), as well as non-autonomous short interspersed terminal repeats (SINEs) and SINE–VNTR–Alu (SVA) elements [77,78].

The vast amount of literature demonstrates that retrotransposition has extensive influence on evolution by introducing genomic diversity (Figure 2). Repetitive sequences might mediate recombination between homologous parts of the genome. The integration of TE within the gene areas might lead to alterations in the coding sequence, splicing sites, and poly-A signaling. TEs are also known sources of various ncRNAs [79] and regulatory regions, including alternative promoters [80,81], enhancers [82], or insulators [83]. As such, TE sequences may serve as a landing platform for transcriptional and epigenetic factors altering the activity of neighboring genes [84,85,86]. Nevertheless, active TEs may introduce potentially hazardous insertional mutation, as observed in various human diseases [87], while improper control of TE sequences may lead to transcriptional dysregulation of nearby genes [85,86]. To counteract these risks, the cell introduced numerous genetic and epigenetic mechanisms aimed at TE inactivation. Thus, the vast majority of TEs are inactive, while with evolution, their sequence deteriorates beyond recognition due to the genetic drift [76]. Only a small proportion of TEs are still retrotransposition-competent [88,89,90]. However, their activity may be evoked in specific tissue types (e.g., stem cells [91], cancer [90]), or in response to stimuli (e.g., immune reaction) [89].

TE re-activation in specific tissues or upon stress conditions occurs due to the erasure of silencing epigenetic modifications. This phenomenon is particularly evident during epigenetic reprogramming that is characteristic of early embryonic development. At this time point, the genome becomes vulnerable to retrotransposition, which may introduce either an advantageous feature or harmful mutation [92]. One of the TE control mechanisms depends on the interaction between the TE DNA sequence and paired KRAB–ZNF (Figure 2) [93]. KRAB–ZNFs may bind specific DNA sequences via zinc finger domains and recruit KAP1 (KRAB-associated protein 1) through the KRAB domain [94]. KAP1, a multidomain protein, interacts with other epigenetic factors, including histone deacetylase, H3K9 methyltransferase (SETDB1), and heterochromatin protein 1 (HP1). In stem cells, the complex may also include DNA methyltransferases (DNMT1/3A/3B) that drive DNA methylation [25,86]. Several high-throughput chromatin immunoprecipitation data identified KAP1 and H3K9me3 deposition at various TE families [95,96,97,98], while KAP1 knockdown was shown to upregulate TE activity in ESCs [95,97,98] and, to a lesser extent, in adult tissues [99,100]. The KRAB–ZNF/KAP1 system seems to serve as the first line of TE control upon chromatin decondensation occurring during early developmental stages, at least for some of the TE subclasses. The complex evokes regional heterochromatinization and stable silencing of TEs.

KRAB–ZNFs comprise a large family of clustered, homologous genes that emerged in a common ancestor of coelacanth and tetrapods and evolved rapidly via multiple waves of duplications followed by genetic divergence [96]. The evolution of KRAB-containing genes exhibits a high correlation with the appearance of new retrotransposons, suggesting a host–pathogen interaction aimed at KRAB–ZNF-led epigenetic restriction of new pathogenic TEs [93]. ChIP-seq profiling linked a number of KRAB–ZNF factors to their target TEs [96,101,102]. Jacobs and colleagues provided elegant evidence on KRAB–ZNF and TE co-evolution, corroborating the hypothesis of an “arms race” between host and pathogen elements [102]. Using the ChIP-seq approach, they identified L1P4, L1P5, L1P6, and L1P3 as target LINEs bound by ZNF93, a stem cell-specific KRAB–ZNF. Interestingly, certain L1P3, as well as younger L1P2 and L1H TEs lacked a 129 bp fragment within 5’ UTR site, which prevented ZNF93-mediated TE silencing. It was demonstrated that ZNF93 co-evolved with L1 elements, from L1P6 to L1P3, to acquire the highest specificity for L1P3. However, a small fragment deletion allowed L1P3-129bp to evade the repression by ZNF93 and initiate a new retrotransposition wave [102]. The arms race component of TE and KRAB–ZNF coevolution corroborates the notion that epigenetic modifications may serve as a time-buyer for the genome [1] before the emergence of a mutation that stabilizes the adaptation to the stimulus. With such a mechanism, the arms race leads to a greater repertoire of KRAB–ZNF genes, TEs, and their associated regulatory regions. As such, TE spread, and the appearance of new repressive KRAB–ZNF shapes the epigenomic signature of the cell by expanding the areas with repressive epigenetic code, such as histone deacetylation, high H3K9me3 level and DNA hypermethylation.

Further studies on the co-evolution of KRAB–ZNFs and TEs refined the arms-race hypothesis. The current model suggests that KRAB-ZNFs not only serve as TE repressors but also as the factors participating in the domestication of TE regulatory sequences [86,103,104]. High-throughput ChIP-exo profiling of the binding sites of 222 KRAB–ZNFs [96] confirmed the ongoing co-evolution between KRAB–ZNF genes and TEs, although the majority of KRAB–ZNF-suppressed TEs were found completely retrotransposition incompetent. KRAB–ZNFs were demonstrated to bind in the vicinity of various transcription factor binding sites. When overlaid with chromatin states obtained from the NIH Roadmap, TE sequences identified as KRAB–ZNF targets associated either with the heterochromatin mark (H3K9me3) or active enhancer marks (H3K4me1/H3K27ac) in a manner dependent on the investigated cell type. Of note, some more ancient KRAB–ZNFs adapted novel functions, thus escaping the arms-race. These observations indicate that KRAB–ZNF and TE co-evolution is linked not only to host–pathogen interactions but suggest the co-option of TE sequences for the epigenetic regulation of the tissue-specific expression program (Figure 2) [96]. Indeed, further studies utilizing a complementary combination of various high-throughput epigenomic profiling (including ATAC-seq, ChiA-PET, and ChIP-seq) provided compelling evidence supporting the TE domestication model. Pontis and colleagues demonstrated that certain classes of young TEs are epigenetically de-repressed during zygotic genome activation at the pre-implantation stage [85]. These TEs contributed with their enhancer sequences to increased transcription of the genes involved in early developmental processes. Parallel overexpression of a few KRAB–ZNFs led to TE suppression later on at the ESC stage. Upon tissue speciation, certain TE-derived enhancers might become again de-repressed to facilitate the establishment of a lineage-related expression program [85]. Also, other reports demonstrated that KRAB-ZNF/KAP1-mediated silencing of TE may still be required in some differentiated tissues, e.g., during neural differentiation, or may participate in tissue-specific control of mRNA and ncRNA transcription dependent on TE regulatory sequences [101,105].

Altogether these data suggest that TE-derived enhancers play an essential role in the species speciation and tissue-specific re-wiring of the transcriptional network in stem cells and differentiated cell lineages [85]. Such a feature allows more extensive plasticity and is particularly useful in tissues that require fast adaptation to external stimuli. Indeed, KRAB–ZNF/KAP1 influence on TE regulatory sequences was shown in the cell types with dynamic expression programs, including stem cells [85], neurons [99], and immune system [100]. It remains to be tested to what extent such epigenetic regulation is required for appropriate determination and maintenance of cellular fate, as well as whether other DNA activities (e.g., generation and maintenance of topologically associated domains) may be affected by KRAB–ZNF–TE regulatory network.

The current data show a strong connection between genetic mutations within TE regulatory sequences and loss of KRAB–ZNF binding ability. However, it may be also envisaged that epimutations occurring as the evolutionary changes in cytosine methylation status may also impair KRAB–ZNF binding to DNA. Certain KRAB–ZNF factors recognize the motifs harboring a CpG site [25,106,107,108]. It is well established that some KRAB–ZNFs attach to their consensus motifs only in the context of methylated CpG, while an unmethylated cytosine abolishes the interaction. This includes ZFP57 [25] and ZNF445 [107]—the factors involved in imprinting maintenance. A reverse relation (i.e., higher KRAB–ZNF affinity to unmethylated rather than methylated site) was also observed, e.g., in case of mice Zfp568 and its target site residing within placental-specific *Igf2-P0* promoter [108]. Further studies are needed to resolve the question of whether permanent changes in DNA methylation may modulate KRAB–ZNF binding potency, thus re-shaping the KRAB–ZNF target portfolio, including TE target sequences.

## 7. Cancer Microevolution—Focus on Epigenomic Alterations

**Figure 3:**
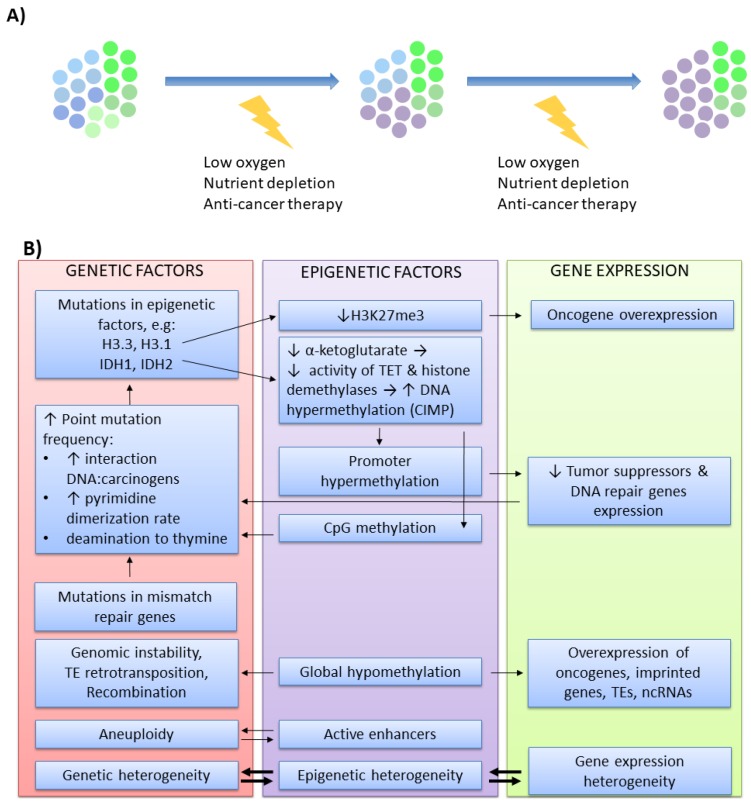
Genomic and epigenomic heterogeneity in cancer. (**A**) Upon (micro)environmental insults (low oxygen, nutrient depletion, anti-cancer drugs), genomic and epigenomic heterogeneity in cancer cell population facilitate the survival and outgrowth of the clones with the features crucial for the adaptation to changing conditions (purple circles). (**B**) The examples of genetic and alterations that affect each other during cancer microevolution.

Another example of interwoven co-evolution between genetic and epigenetic patterns is carcinogenesis. Cancer cells accumulate genetic and epigenetic alterations that provide a selective advantage due to the enhanced proliferation and higher flexibility in response to various challenges (e.g., space restriction, oxygen and nutrient deficiency, anti-cancer therapies). Wider clonal diversity allows escaping selective pressure when the conditions become adverse, resulting in the expansion of the fittest clones (Figure 3A) [109,110]. From such a perspective, carcinogenesis is regarded as a microevolutionary process. For a long time, genetic changes were regarded as the initiators of carcinogenesis. This thesis is based on several observations. It is well established that cancer develops in the carriers of predisposing genetic mutations. Cancer cells are genetically unstable and harbor various DNA mutations that are found in multiple tumors of different origins. Moreover, numerous DNA damaging agents (chemical mutagens, ionizing radiation, viruses) are associated with mutagenesis and cancer development. With the intensive development of the newer field, epigenetics, it is now clear that all these aspects are also valid for epimutations. In comparison to healthy tissues, cancers are epigenetically unstable and have aberrant epigenomic profiles, which may be modified by various chemicals. Moreover, certain epimutations were identified as cancer susceptibility factors [111,112,113,114,115,116]. Cancer epigenomic signature is largely altered when compared to healthy tissues from which it originated [117]. In tumors, the genome becomes globally hypomethylated [118] except for hypermethylation of the hotspots, usually residing within the promoters of tumor suppressor genes and DNA repair genes [119]. The chromatin state is changed due to disturbed 3D chromatin structure, improper functioning of chromatin remodeling enzymes [120,121,122,123], as well as aberrant deposition of histone posttranslational modifications and histone variants within coding, noncoding and regulatory sequences. The expression profile of numerous ncRNAs is also altered [124,125].

While genetic events are well-established triggers of carcinogenesis, the evidence for such a role for epimutation is rarely reported. Nevertheless, they lay the ground for the possibility of cancer development initiated by epigenetic alterations. For instance, experimentally hypermethylated p16Ink4a promoter induced spontaneous tumors in around one-third of epigenetically engineered mice [116]. An increasing amount of data indicate that carcinogenic metals with low mutagenic potential (e.g., nickel [126,127], arsenic [128,129], cadmium [130,131]) affect DNA methylation and posttranslational histone modifications. Germline transmitted DNA hypermethylation responsible for increased cancer risk was identified in the promoters of *MLH1* and *MSH2* in several Lynch syndrome patients [132,133,134], *DAPK1* in chronic lymphocytic leukemia [135], BRCA1 in breast cancer [136] or RB1 in retinoblastoma [115]. A relatively low amount of identified heritable epigenetic traits associated with cancer susceptibility may reflect the complexity of reprogramming mechanisms at play during development that involves global erasure and re-establishment of epigenomic profiles. Of note, some transgenerationally transmitted marks are maintained thanks to genetic variants acting in *cis* or *trans*, and as such, should not be regarded as epimutation [114]. 

Regardless of which alteration occurs first as a cancer initiator—genetic or epigenetic—further modifications within the genomic and epigenomic landscape cooperate (Figure 3B) to provide better adaptation for the growing tumor [125]. An increasing amount of data pinpoints recurrent genetic aberrations in histones or epigenetic modifiers [125,137]. High-throughput profiling approaches demonstrated that specific genetic alterations may affect the epigenome-wide profile. For example, K27M substitution in H3.3 and H3.1 in pediatric brain tumors leads to lowered H3K27me3 deposition [138], which may promote gene activation. *IDH1* and *IDH2* mutations result in a reduced level of α-ketoglutarate, a substrate for TET enzymes and histone demethylases. Impaired TET functioning results in increased DNA hypermethylation, known as CpG island methylator phenotype (CIMP), a phenomenon observed in many tumors [139,140]. Besides, changes within the DNA sequence may also affect a CpG site, as well as non-coding and regulatory regions, thus affecting cancer epigenomic signature [113,125,141]. Inversely, epigenomic aberrations affect the genome. Genome-wide hypomethylation results in transcriptional activation of oncogenes, imprinted genes, transposable elements, and ncRNAs. Low level of methylation impairs genomic stability [142], while increased TE-mediated retrotransposition and recombination frequently disrupt the genes implicated in tumorigenesis [143,144,145]. In contrast, high methylation within the promoter region inhibits the expression of several tumor suppressors and DNA repair genes [146,147]. TE activity and reduced expression of DNA repair genes augment mutagenesis [148]. Moreover, methylated cytosines may provoke direct point mutations more frequently than unmethylated cytosines. Methylated cytosine enables stronger DNA interaction with chemical carcinogens and increases the rate of pyrimidine dimerization evoked by UV light exposure [149]. Cytosines are also prone to deamination to uracil (in the case of unmethylated cytosine) or thymine (in the case of methylated cytosine). The repair mechanisms are less efficient when dealing with mismatched G:T, particularly in highly proliferating cells, which results in frequent C to T substitutions. This is particularly pronounced in tumors with mutated mismatch repair genes (e.g., *MLH1*) [125], which further underlines the cooperation between genetic and epigenetic factors in cancer microevolution. Many mechanistic aspects of such cooperation are relatively well described. Other reported observations on genetic and epigenetic interplay are based mainly on association studies. For example, high-throughput profiling of chromatin states demonstrated that active enhancers correlate with aneuploidy [9]. Furthermore, recent methylome and mutation load profiling in chronic lymphocytic leukemia showed that epigenomic heterogeneity occurs in the clones with increased genetic heterogeneity, particularly in more aggressive, high-risk patients [150]. As these observations are mainly associative, the exact causative relationship between these features requires further examination.

One of the most intriguing questions in cancer epigenomics is the etiology of a unique, cancer-related DNA methylation pattern. The evidence obtained so far indicates that the wide stretches of hypomethylated DNA are associated with global changes in chromatin architecture influenced by a mutation in chromatin modifiers. These mutations may affect DNA methylation and demethylation processes, particularly the functioning of TET and DNMT enzymes [124]. Also, the molecular mechanisms implicated in selective regional hypermethylation of TSG promoters are just beginning to emerge. The most compelling evidence links a large proportion of hypermethylated hotspots to the genomic regions that are bivalent in embryonic stem cells (ESCs) and repressed via the H3K27me3 mark during lineage specialization [151,152,153]. Bivalent domains are transcriptionally inactive, contain both repressive H3K27me3 and activating H3K4me3 marks, while DNA remains hypomethylated. In ESCs, such domains are poised for activation or repression depending on the cell differentiation program [23,154]. It is suggested that developmentally marked H3K27me3-rich regions may be primed for aberrant DNA hypermethylation in cancer cells. In tumors, but not in normal cells, EZH2 (H3K27 methyltransferase) co-localizes with DNMTs at target loci [152]. It is still unclear which cancer-related factors enable such interplay; however, it is likely that an additional repressive mark, H3K9 methylation, may be involved [151]. Recent studies identified a component of NuRD complex, CHD4 (chromodomain helicase DNA-binding protein 4), which is engaged in targeted hypermethylation of TSG promoters. CHD4 is recruited to the lowly expressed genomic regions in response to oxidative DNA damage. Next, it associates with DNMTs, EZH2, and G9a (H3K9 methyltransferase) and mediates transcriptional silencing of the injured site through DNA methylation, H3K27me3 and H3K9me2. Although upon DNA repair majority of these marks may be removed, some may stay, particularly if providing a selective advantage to (pre)cancerous phenotype due to TSG silencing [155]. Interestingly, splicing variants of delta DNMT3B were also shown to confer DNA hypermethylation within specific genomic locations; however, the exact molecular mechanisms for such selectivity remain unknown [156]. Another hypothetical mechanism for targeted TSG hypermethylation may depend on KRAB–ZNF factors associated with particular cancers [157,158]. As it was mentioned previously, KRAB–ZNFs bind to specific DNA sequences and mediate deposition of repressive H3K9me3 mark, histone deacetylation, and in ESCs—mitotically heritable DNA methylation [159]. While the expression of multiple KRAB–ZNFs becomes deregulated in various tumors [157,158], it remains to be tested whether KRAB–ZNFs may also participate in the sequence-specific DNA hypermethylation, particularly in the cells with stem cell-like phenotype. 

The stepwise process that shapes the epigenomic and genomic landscape in cancer cells remains unclear. Epigenetic alterations are far more frequent than genetic events, and in contrast to mutations, they are reversible. This renders epigenetic mechanisms more flexible, and therefore, more suitable for prompt adaptation to adverse microenvironmental conditions. Indeed, the epigenomic landscape of the cancer cells exposed to acute conditions frequently undergoes reprogramming that may help survive the challenge. For example, hypoxia impairs TET functioning, which results in higher DNA methylation within TSG promoters [160]. On the contrary, in another experimental setup, hypoxia evoked global loss of methylation in cancer cells [161]. Of note, many hypoxia and epigenetic pathways are interwoven, as epigenetic modifiers may posttranslationally modify hypoxia-responsive genes affecting their stability. In turn, the hypoxia response regulates the expression of the genes involved in epigenetic signaling [161,162,163]. Epigenetic modifications also play an important role in the acquisition of resistance to various therapies [164,165], including adoptive cell therapy [166]. Indeed, clonal methylome profiling in glioma revealed that epigenomic heterogeneity plays an essential role in chemo- and radiotherapy resistance [167]. Wylie and colleagues tested melanoma and mesothelioma cell explants resistant to in vivo adoptive cell therapy due to antigen silencing. Upon treatment with DNMT inhibitors, a proportion of the explanted cells restored the expression of various immunogenic antigens [166]. These observations indicate that epigenetic divergence is essential for drug sensitivity and resistance. From the clinical point of view, profiling epigenomic states and their evolution may play a vital role in predicting and monitoring therapy outcomes. Moreover, reversing cancer-related or therapy-resistant epigenomic signature with epidrugs may improve current designs of anti-cancer treatment options.

## 8. Conclusions and Future Perspectives

Novel methods of investigating epigenomes are still expanding. The enormous amount of data emerging from high-throughput technology studies resolves many questions about different aspects of human biology. The DNA methylation profile or histone posttranslational modifications of an individual contain a plethora of information about the development, health, disease, and even evolution of our species. The data indicate that many epigenetic features can be inherited to daughter cells and also to offspring. In specific circumstances, epimutations can overcome epigenetic barriers occurring during development and persist in the next generations. Numerous external environmental factors, as well as genetic alterations, can affect the epigenome, and as such, influence the health outcome of an individual or provide better adaptation to new conditions. In both cases, the plasticity of epigenome can be viewed as a buffer or a time-buyer mechanism that serves as the first line of response to external stimuli, without affecting genetic sequence. The importance of such a mechanism is further highlighted by the fact that such changes can be reversed with the usage of specific compounds affecting different components of the epigenomic landscape, i.e., HDAC or DNMT inhibitors. Deliberate re-shaping of epigenome may be useful in a broad spectrum of human biological processes. The developing epigenome editing technology may provide, in the future, valuable tools for both: precise and global targeted modifications. Therefore, further exploration of epigenetic alterations affecting individuals and potentially their offspring is of great importance.

Whole-genome profiling of chromatin states helps understand the age- and disease-related alterations of the human epigenome. For example, it is possible to estimate human aging rates based on the methylation profile of whole-blood cells [168] or to predict the age of multiple tissues through an “epigenetic clock” composed of selected CpGs [169]. As mentioned above, the unstable epigenome may be an outcome of a combination of genetic mutations and naturally occurring epigenetic changes acquired with age or as a result of injury or external stimuli [48]. Malignant cells acquire multiple genetic and epigenetic modifications, thus providing clonal diversity and increased adaptation possibilities to the unfavorable environment [109]. The subsequent modifications of chromatin structure lead to impaired gene expression underlying tumor heterogeneity.

Much effort has been made to produce global maps of epigenomic states in different cell types, representing health and disease. It is of note, however, that the studies should more frequently take into account the complexity of epigenetic regulation over a single genetic locus, which may include DNA methylation, histone post-translational modifications, and ncRNA expression profile. Moreover, single-cell approaches or utilization of a purified population of particular cell types will improve the interpretation of epigenomic profiling datasets. Although we must still rely on epigenomic global analyses to find the answers relate to epigenetic mechanisms behind observed phenotypes, newly identified candidate genes or genetic regions should be individually tested in the context of a particular disorder. Epigenomic editing with the CRISPR/Cas9 system opens new possibilities towards a more comprehensive portrayal of epigenetic events occurring in various normal and disordered conditions. It is tempting to speculate that future results utilizing high-throughput methods will facilitate the detection of specific markers enabling the identification of tissues that show evidence of accelerated age or disease progression. Moreover, global analyses may provide novel possibilities in prediction, diagnosis, and treatment of complex human disorders.
